# Virtual reality exposure therapy for panic disorder

**DOI:** 10.1192/j.eurpsy.2023.972

**Published:** 2023-07-19

**Authors:** S.-E. Cho, H. Ma, S.-G. Kang

**Affiliations:** Department of Psychiatry, Gil Medical Center, Incheon, Korea, Republic Of

## Abstract

**Introduction:**

Virtual reality exposure therapy (VRET) is a treatment in a virtual environment based on the representation of a patient’s disease in virtual reality. VRET means getting used to a specific situation by intentionally facing the patient’s fearful situation based on the ‘exposure technique.’ By repeatedly exposing the patient to a fearful situation, in the end, inducing the intensity of fear to decrease without avoiding the situation. Virtual reality exposure therapy (VRET) was initially used to treat phobias, anxiety disorders, and stress. It has been proven to be an effective psychotherapy method mainly focusing on acrophobia, flight phobia, arachnophobia, social phobia, and post-traumatic stress disorder (PTSD).

**Objectives:**

Virtual reality exposure therapy (VRET) is used to treat phobias, anxiety disorders, and stress. The virtual reality treatment system can reproduce virtual scenes with the environment modified or removed, and can familiarize patients with such environments. This study aims to verify the degree of improvement of the symptoms of panic disorder by conducting VRET that we have made considering the degree of gradual exposure to the panic disorder group and the control group.

**Methods:**

A total of 60 subjects were included in this study, including 43 patients with panic disorder and 17 control group. Subjects were systematically exposed to specific situations over five steps. We checked the heart rate, body temperature, EEG and symptoms before and after exposure using the following assessment instruments; Subjective Units of Discomfort (SUD), Anxiety Sensitivity Index (ASI), Panic Disorder Severity Scale. Repeated measures ANOVA was performed separately for presence, SUDS (distress) scores, and PDSS across time points.

**Results:**

In the patient group, the program proved to be effective as the sensitivity to anxiety decreased significantly after the VR program (F=3.570, p<0.05). Among the subcategories of ASI, the fear of anxiety symptoms showed statistical significance between sessions (F=3.883, p<0.05) and in the interaction between group and time (F=4.585, p<0.01). The study confirmed the effectiveness of the VR program in situations that mainly induce panic attacks in patients diagnosed with panic disorder (elevator riding, driving a car, driving a car in a tunnel, driving a car in rain). The program effect was significant through repeated measurement ANOVA for each variable of EEG (Alpha, Beta, Delta, Gamma, Theta) (F=3.249, p<0.01).

**Conclusions:**

VRET is the process of gradually being exposed to fear stimuli, deliberately confronting and experiencing a fear situation, and learning that nothing dangerous will happen. Even if the patient experiences bodily sensations that are the subject of fear, they can reduce their fear of bodily sensations by repeatedly confirming that they are safe and getting used to them.

**Disclosure of Interest:**

None Declared

